# Allergic rhinitis, allergic contact dermatitis and disease comorbidity belong to separate entities with distinct composition of T-cell subsets, cytokines, immunoglobulins and autoantibodies

**DOI:** 10.1186/s13223-022-00646-6

**Published:** 2022-02-11

**Authors:** Wenjia Chai, Xuyi Zhang, Meixiong Lin, Zhuo Chen, Xiaolin Wang, Changqing Wang, Aoyan Chen, Caisheng Wang, Hongwu Wang, Honghong Yue, Jingang Gui

**Affiliations:** 1 Laboratory of Tumor Immunology, Beijing Pediatric Research Institute, Beijing Children’s Hospital, Capital Medical University, National Center for Children’s Health, Beijing, 100045 China; 2 Key Laboratory of Major Diseases in Children, Ministry of Education, Beijing Pediatric Research Institute, Beijing Children’s Hospital, Capital Medical University, National Center for Children’s Health, Beijing, 100045 China; 3 Third Medical Center of Chinese, PLA General Hospital, Beijing, 100000 China; 4 Department of Allergy, Inner Mongolia Xilinguo League Central Hospital, Xilinhot, 026000 China

**Keywords:** Allergic rhinitis, Allergic contact dermatitis, Immune responses, T cells, Cytokines, Autoantibodies

## Abstract

**Background:**

Allergic rhinitis (AR) and allergic contact dermatitis (ACD) are prevalent allergic diseases and have significant impacts on patients’ daily life. Despite many studies on AR or ACD have been conducted separately, little is known about the immune responses in patients of AR combined with ACD and the interplay between AR and ACD. Our study compared various aspects of immune elements in patients with AR or/and ACD, aiming to characterize the immune responses in AR, ACD, and AR combined with ACD.

**Methods:**

A total of 57 patients diagnosed with AR or/and ACD and 28 healthy volunteers were included. AR patients were further divided into seasonal AR (SAR) and perennial AR (PAR). All subjects’ blood samples were taken to assess the concentration of immunoglobulins, complement C3, C4, autoantibodies and cytokines in serum by immunoturbidimetry, ELISA or Luminex200 platform. Peripheral blood mononuclear cells (PBMCs) were subjected to the analysis of lymphocyte subpopulations by flow cytometry.

**Results:**

It indicated that AR disease caused elevated levels of IgE, IgA, IgG, IgG4, as well as IL-4, IL-15, IL-8 and IL-6 in serum. AR patients possessed a decreased CD4/CD8 ratio and an increased proportion of memory CD4 + T-cell subset, with a skewed Th2 response and an enhanced CD8 + T-cell activation. Compared with patients with sole AR or ACD condition, AR + ACD patients presented with a significantly increased proportion of memory CD8 + T-cell subset and were prone to autoimmune disorders as indicated by the increased autoantibodies. The immune elements in patients with ACD only were least affected compared with those in other conditions. Additionally, seasonal or perennial AR patients exhibited different cytokine profiles and proportions of memory T-cell subsets.

**Conclusions:**

In this study, we illuminated the respective characteristics of immune responses in AR, ACD, and AR combined with ACD. Meanwhile, we discovered that the PAR and SAR patients possessed different cytokine profiles and T-cell compartments. It suggested that these allergic conditions belong to different disease entities. Characterizing the detailed immune changes in these allergic diseases would help to develop proper treatments targeting particular immune elements in different allergic diseases.

**Supplementary Information:**

The online version contains supplementary material available at 10.1186/s13223-022-00646-6.

## Background

Allergic rhinitis (AR) and allergic contact dermatitis (ACD) are common allergic diseases worldwide characteristic of high IgE and allergic inflammation readily triggered by allergens [1, 2]. AR is the most prevalent mucosal inflammation condition generally manifested with sneezing, nasal congestion, nasal itching and rhinorrhea [1]. It is believed that AR is closely related to the inhaled allergen, while ACD is directly connected with skin exposure [2]. Clinical presentations of ACD in acute phase include pruritus, dryness, erythema and scaling. It can develop into chronic inflammatory conditions upon continuous allergen exposure, and the exact mechanism remains unknown [3, 4]. The diagnoses of AR and ACD mainly depend on the occurrence history, regular clinical laboratory tests, skin prick tests and epicutaneous patch tests [2, 5]. Avoidance of allergens is one of the crucial measures for restraining atopic conditions of AR and ACD. While traditional drugs such as antihistamines and corticosteroids can relieve the symptoms, there is no known cure with only the allergen-specific immunotherapy (AIT) lighting a hope for complete remission [2, 6]. AR and ACD seriously influence patients’ life quality and bring economic burden to patients and society [7]. Therefore, it is meaningful to investigate the exact immunological characteristics and the differences between AR and ACD, and to explore the mutual interplay between them when patients are inflicted with AR together with ACD.

AR is an IgE-mediated type I hypersensitivity process that involves several types of immune cells and cytokines [8, 9]. Depending on its sensitization to cyclic pollens or year-round allergens, AR has been classified as seasonal allergic rhinitis (SAR) or perennial allergic rhinitis (PAR) [1]. Symptoms of AR are triggered by allergens, and antigen-specific IgE is produced as a result of complex interactions between dendritic cells, B cells, T cells, mast cells, and basophils [9, 10]. Along with antigen presentation and Th2 polarization of T cells, several critical cytokines such as IL-4, IL-5 and IL-13 are produced [11]. The allergen-specific Th2 response subsequently induces B-cell differentiation and class-switch towards IgE-producing plasma cells mediating inflammatory responses [11, 12]. Further studies demonstrated that in nasal secretions, various cytokines including IL-4, IL-1, IL-2, IL-6, GM-CSF and TNF-α were implicated in AR development and progress [13, 14]. In addition, Treg cells, characterized by the production of anti-inflammatory cytokines such as IL-10 and TGF-β, are likely to be important in the control and resolution of AR [15–17]. There is a growing appreciation that AR is not only a disease restricted to nasal passages, but also a manifestation of systemic airway disease [6]. It will be meaningful to investigate the immune process in peripheral blood in addition to nasal secretions.

By contrast, ACD is a delayed type IV hypersensitivity reaction resulting from the activation of allergen-specific T cells [18]. ACD occurs in the dermis and epidermis, where specific T cells are activated by hapten and an inflammatory cascade is triggered [18]. In light of the knowledge from studies on AR and ACD, it has been accepted that they share similarities of many aspects in their pathogenesis and immune responses. For instance, their allergic inflammatory presentations are driven by multiple immune pathways, especially T-cell mediated immunity [19, 20]. Nevertheless, it remains elusive whether AR would march faster or become worse from the immunological perspective when ACD is accompanied, or vice versa. In other words, it is not known yet if these two conditions would interplay and reshape the immune response reciprocally, or they merely belong to two independent entities. Through dissecting various immune features of patients with AR/ACD only or AR concomitant with ACD, our study aims to find the clue to the relationship, if any, between these two highly frequent atopic conditions.

## Methods

### Patients

The present study recruited 57 patients diagnosed with AR and/or ACD and 28 healthy volunteers aged from 19 to 35 years. The information of the patients and healthy controls was displayed in Table 1. All subjects, including healthy volunteers, were subjected to skin prick tests and epicutaneous patch tests. Diagnosis of AR was based on medical history, clinical laboratory findings and skin prick tests according to ARIA guidelines [5]. Based on the results from skin prick tests on cyclic pollens or year-round allergens (summarized in Table 2), AR patients were divided into SAR or PAR. Diagnosis of ACD was made according to medical history, physical examination and confirmed by the epicutaneous patch tests. AR combined with ACD patients were defined as the AR + ACD group. The study was approved by the Medical Ethics Committee of Third Medical Center of Chinese PLA General Hospital and Inner Mongolia Xilinguo League Central Hospital. Written consents were provided by all participants.

**Table 1:** Patient information

Group	Gender (female/male, n)	Age (years ± SEM)
HC (n=28)	12/16	25.57±1.01
AR (n=30)	12/18	27.93±0.8
ACD (n=12)	6/6	24.17±1.19
AR+ACD (n=15)	6/9	26.53±1.32

**Table 2:** Results of skin prick tests

Allergen		Positive (n, %)			
		HC (n = 28)	AR (n = 30)	AR+ACD (n = 15)	ACD (n = 12)
Mite	*D. pteronyssionus*	0	11, 36.7%	7, 50%	0
	*D. farinae*	0	11, 36.7%	7, 50%	0
Trees pollen	Common silver birch	0	4, 13.3%	2, 13.3%	0
	Mountain juniper	0	18, 50%	7, 50%	0
Weeds pollen	Mugwort	0	2, 6.7%	3, 20%	0
	Japanese Hop	0	5, 16.7%	4, 26.7%	0

### Skin prick test and epicutaneous patch tests

Skin prick tests were performed for the common inhalant allergens in China, including dust mite (*Dermatophagoides. pteronyssionus, Dermatophagoides. farinae*), trees pollen (Common silver birch, Mountain juniper), and weeds pollen (Mugwort, Japanese Hop). All extracts were prepared from the Allergen Manufacturing and Research Center (Peking Union Medical College Hospital, Beijing, China). Histamine phosphate (0.01 mg/ml) was used as the positive control, and an allergen diluent (normal saline solution) as the negative control. The results were evaluated 15 min after application. A wheal diameter ≥ 5 mm after subtracting the negative control for each of the allergens tested was considered as a positive response. The results were listed in Table 2.

The epicutaneous patch (manufactured by Rainmix Biotechnology, Anhui, China) tests were performed on the upper back and evaluated after 48 and 72 h. The test reactions were graded from no reaction to grade + , +  +, and +  +  +, depending on the intensity of the reaction following the recommendations of the International Contact Dermatitis Research Group [21]. The results were listed in Table 3.

**Table 3:** Results of epicutaneous patch tests

Allergen^a^	Positive (n, %)			
	HC (n = 28)	AR (n = 30)	ACD (n = 12)	AR + ACD (n = 15)
Potassium dichromate	0	0	3, 25%	2, 13.3%
Ursol	0	0	5, 41.7%	5, 33.3%
Paraben mix	0	0	3, 25%	0
Cobalt chloride	0	0	3, 25%	1, 6.7%
Nickel sulfate	0	0	1, 8.3%	4, 26.7%
Colophony	0	0	1, 8.3%	1, 6.7%
Fragrance mix	0	0	0	2, 13.3%
Thiomersalate	0	0	0	2, 13.3%
Quaterium-15	0	0	0	3, 20%
P-tert-butyl phenol formaldehyde resin	0	0	0	1, 6.7%

^a^Kathon CG, formaldehyde, bronopol, quadrol, mercaptobenzothiazole, carba mix, rubber mix, N-cyclohexylthiopeptide lipid, quinol and epoxy resin are not listed in the table on account of none of the subjects showed positive reaction for these allergens

### Flow cytometry

For surface marker labeling, 50 μl EDTA anticoagulated blood of each sample was incubated with fluorochrome-conjugated antibodies for 20 min in dark. After that, 450 μl OptiLyse C (Beckman Coulter, USA) was added for erythrocytes lysis. Following 10 min incubation in dark, 50 μl absolute count beads (Biolegend, USA) were added before events of stained cells were acquired with a FACS CantoII flow cytometer (BD Biosciences, USA).

For intracellular labeling, PBMCs were isolated with Ficoll-Hypaque density gradients. After centrifuging at 1000*g* for 20 min at room temperature, the interphase cell layer was carefully transferred into a 15 ml tube. The cell pellet was washed with 10 ml PBS containing 5% FBS and centrifuged at 600*g* for 5 min. For cytokine detection, 2 × 10^6^ cells of each sample were cultured in 10% FBS RPMI-1640 medium (Gibco, USA) with 50 ng/ml of PAM (Sigma, USA), 1 μg/ml Ionomycin (Yeason, China) in the presence of 1 μg/ml Brefeldin A (Yeason, China) at 37 °C cell incubator with 5% CO_2_. Then cells were permeabilized using Fixation/Permeabilization Solution Kit (BD Biosciences, USA) according to the manufacturer’s protocol after surface marker labeling. Cell events were acquired with FACS CantoII flow cytometer (BD Biosciences, USA) and data were analyzed with FlowJo (v10, Tree Star). Following antibodies were used: For surface marker labeling: CD3 APC-H7, CD4 BV421, CD8 APC-R700, CD19 PE-Cy7, CD16 V500, CD56 PerCP-Cy5.5, CD45RA APC, CD45RO FITC. For intracellular labeling: CD4 PerCP-Cy5.5, CD8 PE-Cy7, IL-4 PE, IFN-γ APC, granzyme B FITC. All antibodies were purchased from BioLegend.

### Measurement of serum cytokines

According to the manufacturer’s instructions of the Human Cytokine/Chemokine Panel MILLIPLEX® MAP kits (Cat. No. HCYTOMAG-60K: IFN-γ, IFN-α2, IL-1β, IL-1α, IL-8, IL-4, IL-6, IL-10, IL-12(p70), IL-15, IL-17A, TNF-α) (Merck Millipore, Germany), serum concentrations of multiple cytokines were measured by a Luminex200 platform (Merck Millipore, Germany). In brief, the plate was pretreated with 200 μl wash buffer for10 min. Next, 25 μl serum of each sample, 25 μl assay buffer and 25 μl mixed beads were added to sample wells. The background wells, QC wells and standard wells were added with corresponding reagents following the instructions. The plate was sealed and incubated with shaking at 4 °C overnight. The next day, the plate was washed twice with wash buffer and 25 μl detection antibodies were added for a 1 h incubation at room temperature. After washed twice with wash buffer, each well was incubated with 25 μl Streptavidin Phycoerythrin for 30 min. After two wash steps, 150 μl sheath fluid was added and the data were collected by the Luminex200 platform. All samples were measured in duplicate.

### Measurement of serum immunoglobulin, IgG4, C3, C4 and autoantibodies

Serum IgG, IgA, IgM and C3, C4 were measured by immunoturbidimetry using an automated Beckman Image 800 Immunochemistry System (Beckman Coulter, USA) according to the manufacturer’s instructions. Serum IgE was measured by ELISA using EUROIMMUN Analyzer I (Euroimmun, Germany) according to the manufacturer’s instructions. Serum IgG4 was measured by ELISA (SAB, USA). Serum autoantibodies (nRNP, Sm, SS-A, Ro-52, SS-B, Scl-70, PM-Scl, Jo-1, CENP B, PCNA, dsDNA, NUCL, Histones, rib-P, AMA-M2) were measured by EUROBlotMater and the data were analyzed by EUROBlotCamera (Euroimmun, Germany).

### Statistical analysis

The scatter plots data were represented as the mean ± SEM. Statistical analyses were performed using one-way ANOVA and Tukey’s multiple comparisons test or two-tailed Student *t*-tests (unpaired) in Prism 7.0 (GraphPad Software, USA). Significant differences between groups were represented by *p < 0.05, **p < 0.01, and ***p < 0.001.

## Results

### Differential levels of serum immunoglobulins and cytokines in AR or/and ACD patients

A total of 57 patients diagnosed with AR or/ and ACD aged from 19 to 35 years were included in the study. AR combined with ACD patients were defined as the AR + ACD group. Results for skin prick tests and epicutaneous patch tests were listed in Tables 2 and 3. As expected, all healthy volunteers showed no reaction in response to these allergens.

**Fig. 1:**
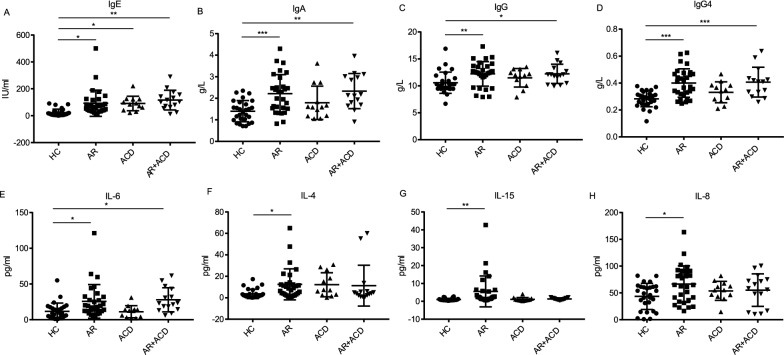
The serum levels of IgE, IgA, IgG, IgG4, IL-6, IL-4, IL-15 and IL-8. **A**–**D** The serum levels of IgE and IgG4 were measured by ELISA, and IgA and IgG measured by immunoturbidimetry. **E**–**H** The concentrations of IL-6, IL-4, IL-15 and IL-8 were determined by the Luminex200 platform. HC, healthy controls, n = 28; AR, allergic rhinitis, n = 30; ACD, allergic contact dermatitis, n = 12; AR + ACD, allergic rhinitis combined with allergic contact dermatitis, n = 15.*p < 0.05, **p < 0.01, and ***p < 0.001 (one-way ANOVA and Tukey's multiple comparisons test)

We firstly investigated the serum levels of immunoglobulin and complement C3, C4. IgE played a key role in type I hypersensitivity mediating various atopic diseases [22]. Indeed, the results showed levels of IgE were elevated in all three patient groups (Fig. 1A). Interestingly, compared with those in HCs, IgA, IgG and IgG4, one of the subclasses of IgG, were elevated in AR group and AR + ACD group, but not in ACD group (Fig. 1B–D). It implied that the IgG and IgA levels were disturbed in AR patients but not in patients afflicted with ACD only. By contrast, the serum IgM and complement C3, C4 were all at comparable levels to controls (Additional file 1: Fig S1A–C).

Next, we examined the levels of serum cytokines (IFN-γ, IFN-α2, IL-1β, IL-1α, IL-8, IL-4, IL-6, IL-10, IL-12(p70), IL-15, IL-17A, TNF-α) in each group by Luminex200 platform. Our data revealed that serum IL-6 remarkably increased in both AR group and AR + ACD group, compared to that in HCs (Fig. 1E). Surprisingly, IL-4, IL-15 and IL-8, three important pro-inflammatory cytokines that involved in Th2 response were found elevated in patients with AR only (Fig. 1D–F), but not in AR + ACD group and ACD group (Fig. 1F–H). Other cytokines were not found to have any difference among different groups (Additional file 1: Fig S1C–J).

### Lymphocytes subsets in the peripheral blood of patients with AR or/and ACD

**Fig. 2:**
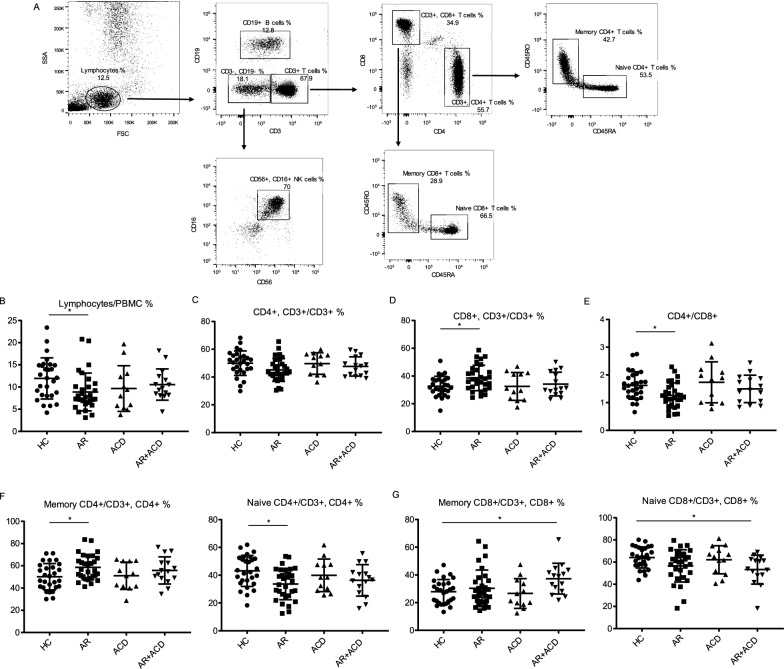
Flow cytometry analysis of lymphocytes subsets in peripheral blood. **A** PBMCs were stained as described in materials and methods. Representative FACS plots of HC and gating strategy were shown. **B** Scatter plots showed the percentage of lymphocytes in PBMC of indicated groups. **C**, **D** Scatter plots showed the percentage of CD4 + or CD8 + T cells in CD3 + T cells of indicated groups. **E** Scatter plots showed the ratio of CD4 + /CD8 + . **F** Scatter plots showed the percentage of naive CD4 + (CD45RA + , CD45RO −) or memory CD4 + (CD45RA − , CD45RO +) T cells in CD4 + T cells. (G) Scatter plots showed the percentage of naive CD8 + (CD45RA + , CD45RO −) or memory CD8 + (CD45RA − , CD45RO +) T cells in CD8 + T cells. HC, healthy controls, n = 28; AR, allergic rhinitis, n = 30; ACD, allergic contact dermatitis, n = 12; AR + ACD, allergic rhinitis combined with allergic contact dermatitis, n = 15. *p < 0.05, **p < 0.01, and ***p < 0.001 (one-way ANOVA and Tukey's multiple comparisons test)

In parallel, the lymphocytes subsets in peripheral blood from indicated groups were investigated. Representative FACS plots of the HC group and gating strategy were shown (Fig. 2A). Compared with HC group, the percentage of lymphocytes was decreased in AR group (Fig. 2B). While CD4 + CD3 + T cells did not show a significant change, an increase in CD8 + CD3 + T cells was observed in AR group, but not in ACD group or AR + ACD group (Fig. 2C, D). As a result, CD4/CD8 ratio was decreased in AR group (Fig. 2E). There was no significant difference observed in CD19 + B cells and CD3-CD56 + CD16 + NK cells among four groups (Additional file 1: Fig S2A, B).

Considering that memory T cells have been observed playing a role in allergic responses [23], we examined the proportion of memory/naive T cell subsets in different patient groups and HCs. An increased proportion of CD45RO + CD45RA-memory CD4 + T cells were observed in AR group (Fig. 2F). Whereas, AR + ACD group presented with an elevated proportion of CD45RO + CD45RA-memory CD8 + T cells (Fig. 2G). The immune cell compartments in patients with ACD only were minimally disturbed. Neither memory CD4 + nor memory CD8 + T cells had any ratio change in ACD patients compared to HCs (Fig. 2F, G).

In sum, AR patients showed the most intense impact on the immune components reflected by an elevation of various serum cytokines (IL-4, IL-15, IL-8, IL-6) as well as a reduced CD4/8 ratio in addition to an augmented memory CD4 + T-cell compartment in the peripheral circulation. AR + ACD group was featured with a moderate change in serum cytokines (increased in IL6 expression only) and an increased ratio in peripheral memory CD8 + T cells. Based on our data, it was perceivable that cytokines and T-cell compartment in patients with ACD only were least affected in comparison with other allergic conditions.

### Decreased IFN-γ + CD4 + /IL-4 + CD4 + cells and increased granzyme B + CD8 + T cells in AR patients

**Fig. 3:**
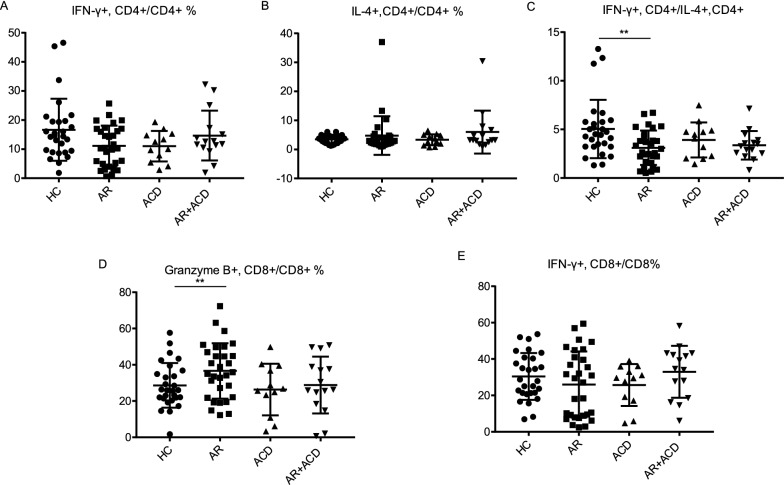
The ratio of IFN-γ + CD4 + /IL-4 + CD4 + was decreased and the proportion of granzyme B + CD8 + T cells was increased in AR patients. **A**–**E** Intracellular cytokine staining was carried out following PMA (50 ng/ml) and ionomycin (1 μg/ml) stimulation for 5 h. Scatter plots showed the percentage of IFN-γ + CD4 + , IL-4 + CD4 + , the ratio of IFN-γ + CD4 + /IL-4 + CD4 + , granzyme B + CD8 + and IFN-γ + CD8 + . HC, healthy controls, n = 28; AR, allergic rhinitis, n = 30; ACD, allergic contact dermatitis, n = 12; AR + ACD, allergic rhinitis combined with allergic contact dermatitis, n = 15. *p < 0.05, **p < 0.01, and ***p < 0.001 (one-way ANOVA and Tukey's multiple comparisons test)

To dissect the T-cell function in these patient groups, intracellular staining for Th1-related cytokine (IFN-γ), Th2-related cytokine (IL-4) and lytic granule (granzyme B) in T-cell subsets were performed. A reduction of IFN-γ + CD4 + subset was observed in AR group (Fig. 3A). Although there was no significant change in the IL-4 + CD4 + subset, the ratio of IFN-γ + CD4 + (Th1)/IL-4 + CD4 + (Th2) showed a decrease in AR group (Fig. 3B, C). Moreover, the proportion of granzyme B + CD8 + T cells was increased while IFN-γ + CD8 + T cells showed no change (Fig. 3D, E). It indicated the imbalance of Th1/Th2 and the activation of CD8 + T cells in AR group.

### Higher incidence and broader spectrum of autoantibodies in AR + ACD patients

Previous research has established that autoantibodies were involved in the pathogenesis of autoimmune diseases [24]. To investigate if the long-term allergic conditions could trigger autoimmunity leading to the production of autoantibodies, we characterized the profile of autoantibodies (IgG) (nRNP, Sm, SS-A, Ro-52, SS-B, Scl-70, PM-Scl, Jo-1, CENP B, PCNA, dsDNA, NUCL, Histones, rib-P, AMA-M2) in the serum of patients. We defined “autoantibody (IgG) positive rate” as the percentage of positive individuals in each group. When a patient showed one or more autoantibody reactivity in the autoantibody profile test, he or she was defined as an autoantibody-positive individual.

**Fig. 4:**
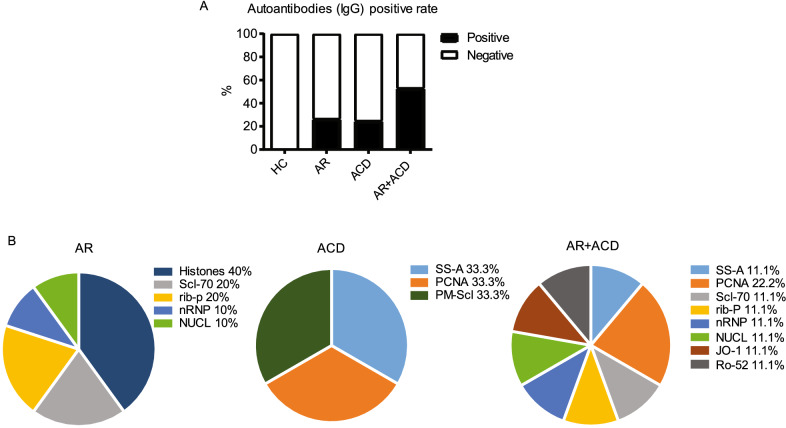
AR + ACD patients showed higher positive rate and broader spectrum in autoantibody (IgG) profile. **A** The percentage of patients with positive reactions to autoantibodies in each group was shown. When a patient showed one or more autoantibody reactivity in the autoantibody profile test, he or she was defined as an autoantibody-positive individual. **B** Autoantibody profile of each patient group was shown. The percentage was calculated as the numbers of seropositivity for each autoantibody compared to the total number of positive reactions of each group

The results showed that the autoantibody positive rate in AR and ACD groups was 26.7% and 25%, respectively (Fig. 4A). Strikingly, in the AR + ACD group, the positive rate was increased to 53.3%, which implied the additive effect of AR and ACD towards autoimmunity (Fig. 4A). As shown in Fig. 4B illustrating the profile of autoantibodies presentation, several patients showed multiple autoantibody specificities. It appeared that the histones IgG was the prevalent autoantibodies in AR patients (Fig. 4B). Moreover, AR + ACD group presented with a more diversified autoantibody spectrum (Fig. 4B). In other words, AR group had a histones autoantibody bias, and AR + ACD group possessed a higher positive rate and a broader spectrum of autoantibodies. Taking together, these results suggested an association of autoimmune propensity with these allergic diseases to various extents.

### Different immunoglobulin levels, cytokines expression and T-cell subsets between PAR and SAR patients

**Fig. 5:**
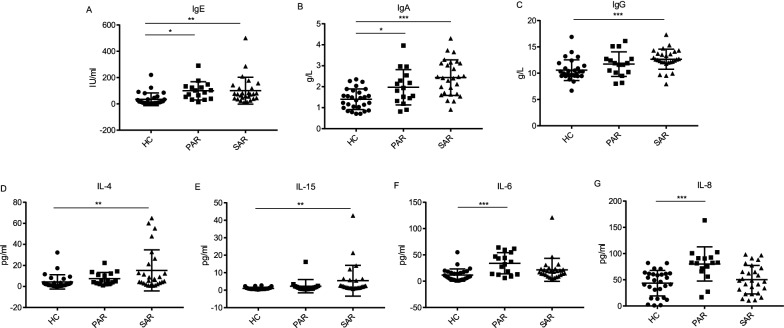
PAR and SAR patients serum exhibited different cytokine profiles. **A**–**C** The serum levels of IgE were measured by ELISA, IgA and IgG were measured by immunoturbidimetry. **D**–**G** The concentration of IL-4, IL-15, IL-8 and IL-6 were determined by the Luminex200 platform. HC, healthy controls, n = 28; PAR, perennial allergic rhinitis, n = 16; SAR, seasonal allergic rhinitis, n = 27.*p < 0.05, **p < 0.01, and ***p < 0.001 (one-way ANOVA and Tukey’s multiple comparisons test). *p < 0.05, **p < 0.01, and ***p < 0.001 (one-way ANOVA and Tukey’s multiple comparisons test)

**Fig. 6:**
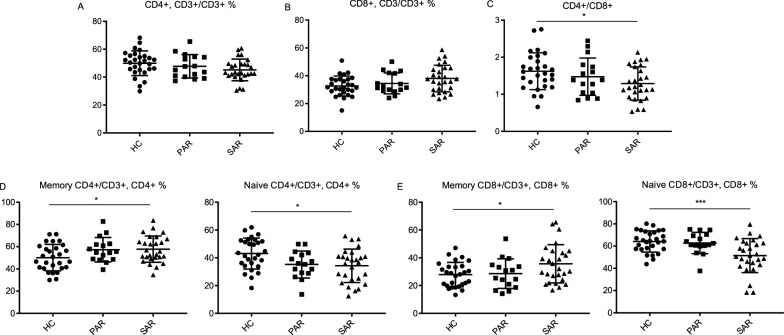
SAR patients showed significant changes in the proportion of T cell subsets. **A**, **B** Scatter plots showed the percentage of CD4 + or CD8 + T cells in CD3 + T cells of indicated groups. **C** Scatter plots showed the ratio of CD4 + /CD8 + . **D** Scatter plots showed the percentage of naive CD4 + (CD45RA + , CD45RO −) or memory CD4 + (CD45RA − , CD45RO +) T cells in CD4 + T cells. **E** Scatter plots showed the percentage of naive CD8 + (CD45RA + , CD45RO −) or memory CD8 + (CD45RA − , CD45RO +) T cells in CD8 + T cells. HC, healthy controls, n = 28; PAR, perennial allergic rhinitis, n = 16; SAR, seasonal allergic rhinitis, n = 27. *p < 0.05, **p < 0.01, and ***p < 0.001 (one-way ANOVA and Tukey's multiple comparisons test)

Considering AR group exhibited significant differences in several cytokines and T-cell subsets compared with HC group, a more detailed analysis was made. AR patients were divided into SAR (n = 27) or PAR (n = 16) depending on the reactivity of skin prick tests to cyclic pollens or year-round allergens (mite) (Table 2). Two patients exhibiting positive reactions to both cyclic pollens and mite were excluded. Data segregation for AR patients revealed that total serum IgA and IgE levels were elevated in both SAR and PAR patients, while total IgG level was only increased in SAR patients (Fig. 5A–C). Cytokine analysis showed an increase of IL-4 and IL-15 in the SAR patients, while IL-6 and IL-8 were elevated in PAR patients (Fig. 5D–G). In T-cell subsets analysis, CD4/CD8 ratio was decreased in SAR patients, although no significant change in CD4 + CD3 + T cells or CD8 + CD3 + T cells was observed (Fig. 6A–C). Similarly, compared with the HC group, only SAR patients showed a significant increase of memory/naive ratio in both CD4 + and CD8 + T cells (Fig. 6D, E). In sum, the results suggested that SAR and PAR are two distinguishable allergic conditions with multiple immune elements presenting at different levels.

## Discussion

In this study, we investigated the immune responses in peripheral blood from AR, ACD and AR combined with ACD patients, including serum levels of immunoglobulin, cytokines, the proportion of lymphocytes subsets, as well as autoantibody profile. It expanded our understanding on the pathogenesis of allergic disease and clarified the different characteristics of immune responses among patients diagnosed with AR or/and ACD.

In the serological examination, AR patients and AR + ACD patients exhibited an elevated level of IgE, IgG and IgA compared with the HC group, while patients with ACD only showed an increase in IgE level but not IgA and IgG. There is consensus that antigen-specific IgE is crucial for the development of these allergic disorders [25, 26]. Contradicted to the belief that IgE is not an important mediator in ACD, the increased IgE in patients with ACD might be a sequela from a vicious circle of sensitization to other allergens due to repeated skin barrier disruption [27]. During the late phase of allergic process, IgE is released and binds to high-affinity receptor FcεRI on the surface of mast cells and basophils [10, 26]. Following allergens binding to allergen-specific IgE, a complex cascade of mediators releasing is triggered [10, 26]. By contrast, allergen-specific IgG, which is also induced by allergen, can downregulate IgE-mediated anaphylaxis by masking allergens and crosslinking with FcεRI and FcγRIIb [28, 29]. On the other hand, IgG could induce an allergic process by activating FcγRs on different cell types when allergen levels are high [30]. The elevation of serum IgG was also found in the mouse model of AR disease [31], besides, the nasal IgG was higher in AR patients compared with healthy controls [32]. There are four subclasses of IgG (IgG1, IgG2, IgG3 and IgG4). The inhaled allergens were strongly associated with serum IgG4 response [33]. Production of IgG4 in allergen-specific immunotherapy (AIT) is an immunological hallmark for successful tolerance establishment [34]. During AIT, AR patients showed a higher increase in serum IgG4 compared to patients with asthma symptoms [35]. Similarly, previous studies found a higher IgG4 level in nasal secretion and serum of AR patients [36, 37]. The fact that IgG4, an immunosuppressive mediator, increased in AR and AR + ACD patients implied that a complex negotiation between allergic inflammation and tolerance was established in these patients. IgA mainly occurs as a monomer form in serum, and it is found as secretory IgA in secretion [38]. It has been reported that the salivary and nasal but not serum IgA levels were increased in AR patients [39, 40]. Deficiency in IgA may cause a change in the mucosal defense preceding the onset of allergy [41]. Other studies reported that a high level of IgA may be associated with mitigation of allergic symptoms, while other data indicated that allergic-specific IgA induced eosinophil degranulation [42–44]. The role of IgA in allergic disorders remains unclear. Our results found remarkably increased IgG and IgA levels in AR and AR + ACD patients, but not in the ACD patients. Based on our multiple measurements on immunological elements, we boldly believe that AR has a much intensive impact on systemic immunity while ACD’s influence is more locally constrained.

In cytokines/chemokines determination, AR patients expressed elevated levels of IL-4, IL-15, IL-8 and IL-6, while AR + ACD patients only showed an increase in IL-6. However, patients with ACD did not show any significant change in these cytokines. IL-4 is a characteristic Th2 cytokine critical for IgE-mediated inflammatory response [45]. IL-6 is a typical pro-inflammatory cytokine [11, 12]. IL-8 is a chemotactic cytokine for neutrophils and primed eosinophils [46]. IL-15 has been reported to be induced by allergen-specific Th2 cells [47]. It plays important roles in T cell activation and homeostasis, survival of B cells, mast cells and eosinophils [48, 49]. Meanwhile, it has been documented that in the nasal lavage fluid of AR patients, the expression of IL-4 and IL-6 increased [50, 51]. Additionally, there is evidence showing that IL-15 prevents AR through the reactivation of antigen-specific CD8 + cells [52]. Likewise, in a context of a more intensive impact on system immunity in AR versus the other two allergic conditions, multiple cytokine changes were found only in AR patients. ACD or comorbidity of AR and ACD seemed a different disease entity from AR based on the cytokine expression profile. Increased IL-6 in AR + ACD patients is possibly an intermediate responder along with the allergy march from ACD to ACD + AR. This conjecture was consolidated by the fact that increased IL-6 in AR mainly occurred in PAR patients, a condition with milder inflammation than SAR [53, 54]. Why PAR patients are the major contributors to IL-8 elevation in the AR group is hard to explain. Our impression is that PAR patients retained a chronic inflammation rather than an acute response in SAR due to their long-term allergen exposure.

The presence of autoantibodies in serum reflects leakiness of central and/or peripheral tolerance and may lead to the manifestation of autoimmune diseases [24]. A plethora of evidence has suggested that serum autoantibodies participate in the progress of allergic diseases [55, 56]. We confirmed that a higher incidence of AR patients presented with autoantibodies in blood samples, with histones as the prevalent antigen, which is congruent with previous studies in animal models [55]. Worth to mention, AR + ACD patients showed higher incidence and more diversified autoantibodies than patients with sole AR or sole ACD. It suggested that comorbidity of AR and ACD poses the patient higher risk for autoimmune disorders.

In lymphocytes subsets analysis, a significant decrease of CD4 + /CD8 + ratio in AR patients was observed. Several previous studies showed no significant difference in CD4 + T cells in AR patients, but some others found increased CD4 + T cells [57–59]. It might own to different allergens or the severity of the disease. Allergic inflammation is mainly driven by Th2 cells [9]. IL-4 is a typical Th2-cytokine, and IFN-γ can be produced by Th1 cells [60]. We found a decrease in IFN-γ + CD4 + /IL-4 + CD4 + ratio in AR patients, which indicated the imbalance of Th1/Th2 in AR. Besides, AR patients showed an increase in memory CD4 + T cells and a decrease in naïve CD4 + T cells, while AR + ACD patients showed similar changes in CD8 + T cell subsets. The allergic condition lead to the formation of a pool of memory allergen [19], while AR and AR + ACD had different preferences in the induction of CD4 + and CD8 + T cells. Moreover, SAR patients exhibited significant changes in memory/naïve subsets of both CD4 + and CD8 + T cells while this was not true for PAR patients. It might be a result from a more pronounced allergenicity in cyclic pollens SAR patients encountered.

## Conclusions

In conclusion, our study characterized the immune responses in AR, ACD and AR combined with ACD. AR patients showed elevated serum levels of IgE, IgA, IgG, IgG4, as well as IL-4, IL-15, IL-8 and IL-6. Furthermore, IL-4 and IL-6 were elevated in SAR patients, while IL-6 and IL-8 were elevated in PAR patients, which hinted the different tendencies of immune responses. What’s more, AR disease caused a decreased CD4/CD8 ratio and an imbalance in T cell subsets, including the increased proportion of memory CD4 + T cells, skewed Th2 response, and enhanced CD 8 + T cells activation. SAR but not PAR caused intense changes in T cell subsets. Despite not intensifying the immune responses, comorbidity of AR with ACD presented with an increased proportion of memory CD8 + T cells and an increased propensity for autoimmune disorders.

## Supplementary Information

**Additional file 1**: **Fig. S1**. The serum levels of IgM, C3, C4 and cytokines. **Fig. S2. **Flow cytometry analysis of B cell and NK cell subsets in peripheral blood.

## Data Availability

The datasets used and/or analyzed during the current study are available from the corresponding author on reasonable request.

## Abbreviations

AR: Allergic rhinitis
ACD: Allergic contact dermatitis
Ig: Immunoglobulin
PBMC: Peripheral blood mononuclear cells
IL: Interleukin
IFN-γ: Interferon-γ
ANOVA test: Analysis of variance test
