# Supplementary material for: Allergic rhinitis, allergic contact dermatitis and disease comorbidity belong to separate entities with distinct composition of T-cell subsets, cytokines, immunoglobulins and autoantibodies
Source: Allergy Asthma Clin Immunol. 2022 Feb 11;18:10. doi: 10.1186/s13223-022-00646-6 (PMC8840545; doi:10.1186/s13223-022-00646-6)
Supplement: Supplementary file 1 — Additional file 1: Fig. S1. The serum levels of IgM, C3, C4 and cytokines. Fig. S2. Flow cytometry analysis of B cell and NK cell subsets in peripheral blood. [file 13223_2022_646_MOESM1_ESM.pdf]

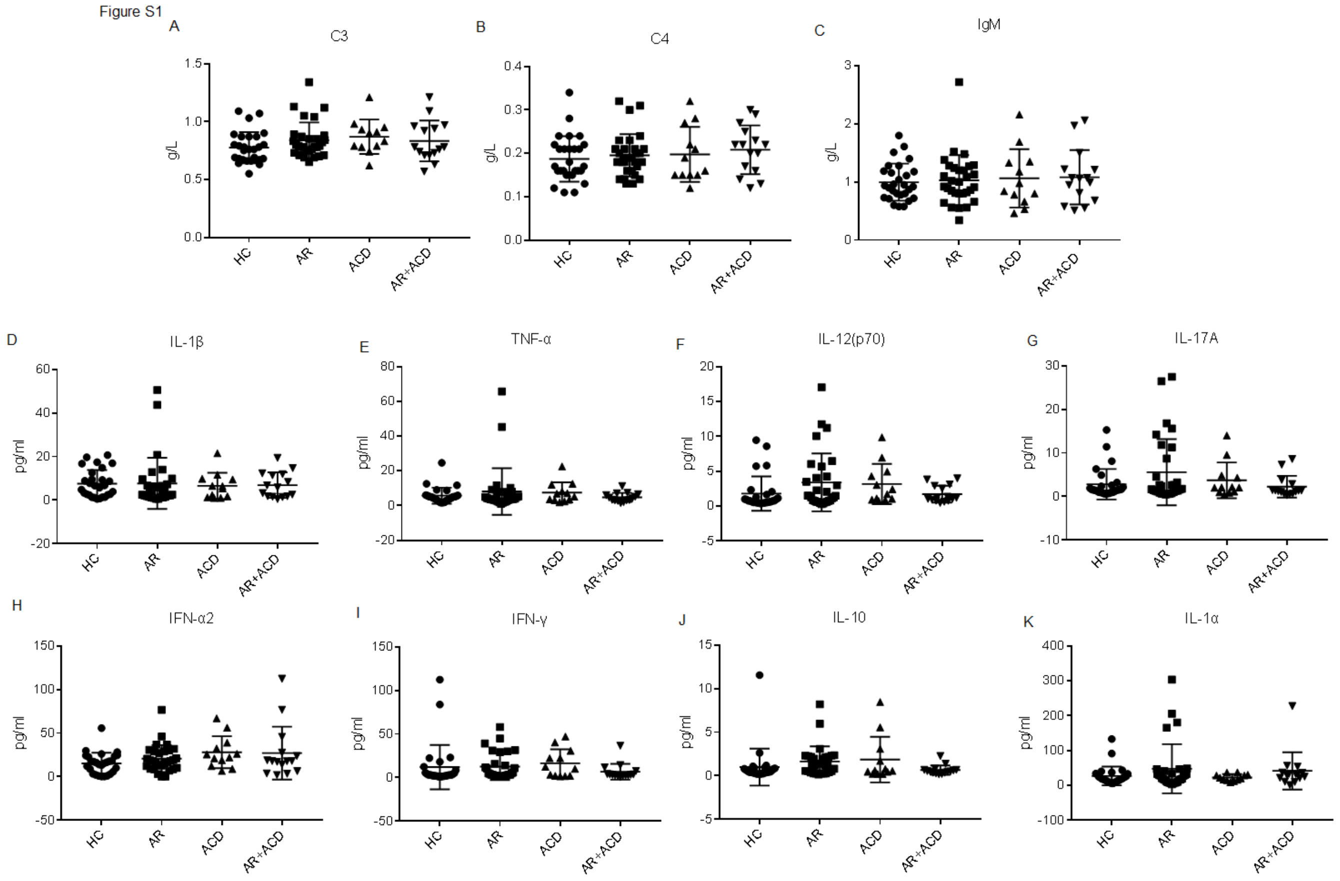

Figure S1. The serum levels of IgM, C3, C4 and cytokines. (A-C) The serum levels of C3, C4 and IgM were measured by immunoturbidimetry. (D-K) The concentration of indicated cytokines were determined by Luminex200 platform. HC, healthy controls, n=28; AR, allergic rhinitis, n=30; ACD, allergic contact dermatitis, n=12; AR+ACD, allergic rhinitis combined with allergic contact dermatitis, n=15. *p< 0.05, **p< 0.01, and ***p< 0.001 (one-way ANOVA and Tukey's multiple comparisons test).

Figure S2

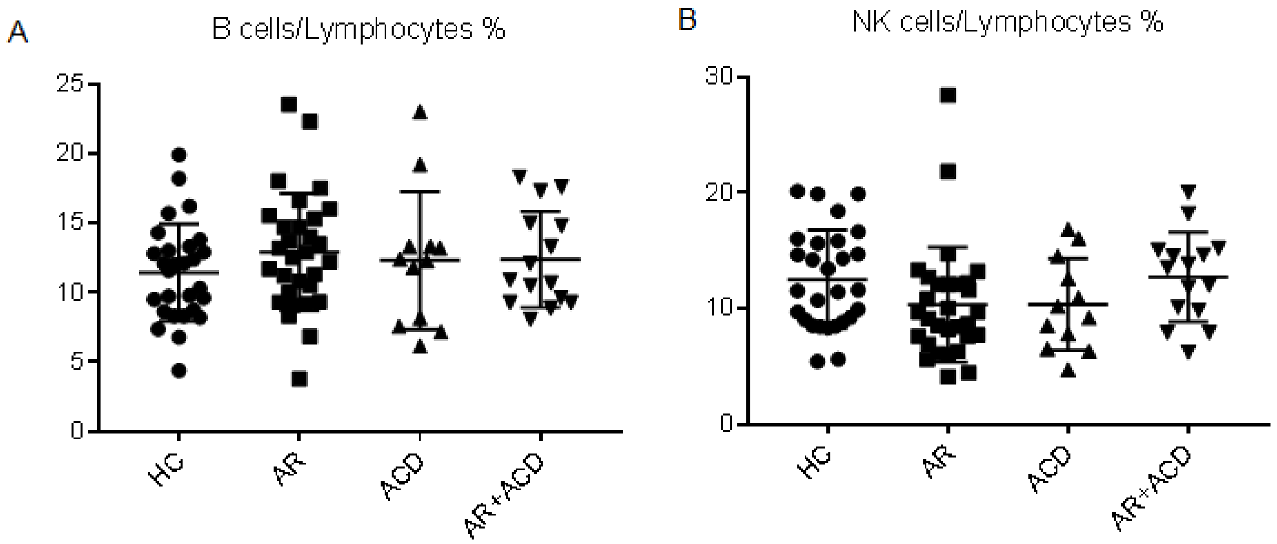

Figure S2. Flow cytometry analysis of B cell and NK cell subsets in peripheral blood. (A, B) Scatter plots showed the percentage of CD19+B cell or CD56+CD16+ NK cell in PBMC of indicated groups. HC, healthy controls, n=28; AR, allergic rhinitis, n=30; ACD, allergic contact dermatitis, n=12; AR+ACD, allergic rhinitis combined with allergic contact dermatitis, n=15. *$p < 0.05$, **$p < 0.01$, and ***$p < 0.001$ (one-way ANOVA and Tukey's multiple comparisons test).
